# Supplementary material for: Pollinator identity and behavior affect pollination in kiwifruit (Actinidia chinensis Planch.)
Source: PeerJ. 2022 Jun 9;10:e12963. doi: 10.7717/peerj.12963 (PMC9188772; doi:10.7717/peerj.12963)
Supplement: Supplemental Information 1 [file peerj-10-12963-s001.docx]

Supplementary Table 1: Pollen grains deposited by insect taxa visiting female kiwifruit cultivar Actinidia chinensis var. deliciosa ‘Hayward’ flowers after foraging on either male or female flowers. Control flowers were held near male or female flowers between 2013 and 2015. Values are means ± standard error (SE); values with no SE are single observations. Letters are based on the Dunn test for male to female pollen transfer for insects with > 5 observations.

| Insect | n | without  pollen | male → female | female → female | group |
| --- | --- | --- | --- | --- | --- |
| Hymenoptera |  |  |  |  |  |
| *Apis mellifera* | 224 | 37.5% | 15,552 ± 4,428 | 924 ± 153 | ab |
| *Bombus hortorum/ruderatus* | 17 | 11.8% | 26,381 ± 13,889 | 0 | a |
| *Bombus terrestris* | 31 | 22.6% | 10,407 ± 2,815 | 4,834 ± 3,442 | a |
| *Lasioglossum* spp. | 24 | 41.7% | 1,955 ± 813 | 1 | abc |
| *Leioproctus* spp. | 21 | 19.0% | 11,400 ± 5,078 | 796 | ab |
| Diptera |  |  |  |  |  |
| *Dilophus nigrostigma* | 42 | 19.0% | 13,402 ± 6,720 | 1,169 ± 416 | ab |
| *Eristalis tenax* | 27 | 14.8% | 4,052 ± 2,970 | 1,680 ± 908 | abc |
| *Helophilus hochstetteri* | 22 | 18.2% | 12,718 ± 6,776 | 0 ± 0 | abc |
| *Melangyna novaezelandiae* | 29 | 34.5% | 759 ± 337 | 190 ± 134 | bcd |
| *Melanostoma fasciatum* | 15 | 53.3% | 4,324 ± 3,837 | 318 | bcd |
| Coleoptera |  |  |  |  |  |
| *Calliprason pallidus* | 4 | 25.0% | 1,115 ± 1,107 | 4 | - |
| *Zorion guttigerum* | 46 | 50.0% | 2,193 ± 1,635 | 417 ± 290 | cd |
| Control | 119 | 70.6% | 71 ± 40 | 0 ± 0 | d |

Supplementary Table 2: Flower handling time for insects foraging on kiwifruit cultivar Actinidia chinensis var. deliciosa ‘Hayward’ in 2013–2015. Values are means ± standard error (SE); values with no SE are single observations. Letters are based on the Dunn test for flower handling time for insects with >1 observation.

| Insect | n | Time (s) | group |
| --- | --- | --- | --- |
| Hymenoptera |  |  |  |
| *Apis mellifera* | 86 | 24.3 ± 1.9 | bcd |
| *Bombus ruderatus* | 12 | 20.6 ± 6.7 | cd |
| *Bombus terrestris* | 20 | 16.4 52.7 | d |
| *Lasioglossum* spp. | 6 | 152.7 ± 89 | abc |
| *Leioproctus* spp. | 12 | 92.7 ± 38.9 | abcd |
| Diptera |  |  |  |
| *Calliphora stygia* | 1 | 76 | - |
| *Dilophus nigrostigma* | 3 | 140.1 ± 65 | ab |
| *Eristalis tenax* | 9 | 116.0 ± 58.5 | abcd |
| *Helophilus hochstetteri* | 7 | 180.0 ± 82 | ab |
| *Helophilus seelandicus* | 1 | 140 | - |
| *Melangyna novaezelandiae* | 9 | 120.8 ± 43.1 | abc |
| *Melanostoma fasciatum* | 4 | 118.5 ± 1.1 | a |
| *Oxysarcodexia varia* | 1 | 175 | - |
| Coleoptera |  |  |  |
| *Zorion guttigerum* | 6 | 192 ± 62.6 | a |
